# Combined association of insufficient physical activity and sleep problems with healthcare costs: a longitudinal study

**DOI:** 10.1590/1516-3180.2023.0241.R2.25032024

**Published:** 2024-06-17

**Authors:** Kelly Akemi Kikuti-Koyama, Ítalo Ribeiro Lemes, Luana Carolina de Morais, Henrique Luiz Monteiro, Bruna Camilo Turi-Lynch, Rômulo Araújo Fernandes, Jamile Sanches Codogno

**Affiliations:** I Student, Department of Physical Education, Laboratory of InVestigation in Exercise (LIVE), Universidade Estadual Paulista (UNESP), Presidente Prudente (SP), Brazil.; II Assistant Professor, Physiotherapy Department, Universidade Estadual Paulista (UNESP), Presidente Prudente (SP), Brazil; Assistant Professor, Physiotherapy Department, Faculdade Israelita de Ciências da Saúde Albert Einstein (FICSAE), São Paulo (SP), Brazil.; Faculdade Israelita de Ciências da Saúde Albert Einstein, Physiotherapy Department, São Paulo, SP, Brazil; III Student, Laboratory of InVestigation in Exercise (LIVE), Department of Physical Education, Universidade Estadual Paulista (UNESP), Presidente Prudente (SP), Brazil.; IV Assistant Professor, Department of Physical Education, Universidade Estadual Paulista (UNESP), Bauru (SP), Brazil.; V Assistant Professor, Physical Education and Exercise Science Department, Lander University, Greenwood, United States.; VI Associate Professor, Laboratory of InVestigation in Exercise – LIVE, Department of Physical Education, Universidade Estadual Paulista (UNESP), Presidente Prudente (SP), Brazil.; VII Assistant Professor, Laboratory of InVestigation in Exercise – LIVE, Department of Physical Education, Universidade Estadual Paulista (UNESP), Presidente Prudente (SP), Brazil.

**Keywords:** Exercise, Health care cost, Sleep, Public health, Lifestyle, Observational

## Abstract

**BACKGROUND:**

The magnitude of economic losses attributed to sleep problems and insufficient physical activity (PA) remains unclear. This study aimed to investigate the association between insufficient PA, sleep problems, and direct healthcare costs.

**OBJECTIVE:**

To investigate the association between insufficient physical activity (PA), sleep problems, and direct healthcare costs among adults.

**DESIGN AND SETTING:**

Adults aged ≥ 50 years attended by the Brazilian National Health Service were tracked from 2010 to 2014.

**METHODS:**

Direct healthcare costs were assessed using medical records and expressed in US$. Insufficient PA and sleep problems were assessed through face-to-face interviews. Differences were identified using the analysis of covariance and variance for repeated measures.

**RESULTS:**

In total, 454 women and 166 men were enrolled. Sleep problems were reported by 28.9% (95%CI: 25.2% to 32.4%) of the sample, while insufficient PA was reported by 84.8% (95%CI: 82.1% to 87.6%). The combination of sleep problems and insufficient PA explained 2.3% of all healthcare costs spent on these patients from 2010 to 2014, which directly accounts for approximately US$ 4,765.01.

**CONCLUSION:**

The combination of sleep problems and insufficient PA plays an important role in increasing direct healthcare costs in adults. Public health stakeholders, policymakers, and health professionals can use these results to reinforce the need for strategies to improve sleep quality and increase PA, especially in nations that finance their National Health Systems.

## INTRODUCTION

The prevalence of sleep problems (e.g., obstructive sleep apnea, insomnia, and snoring) in adults is high worldwide,^1–3^ which is concerning due to its association with the development of many diseases.^4^ The epidemiological background, which is characterized by a high prevalence of the outcome and an association with diseases, supports a relevant economic burden attributed to sleep problems.^5^ In fact, primary care costs for medicines are 75% higher in Brazilian adults who report severe sleep difficulties than in those who report normal sleep,^6^ while the total healthcare costs related to sleep problems and attributed conditions in Australia reached US$ 655.5 million in 2019–2020.^5^

Similar to sleep problems, insufficient physical activity (PA) is also a common outcome among adults and is associated with the development of many diseases and increased healthcare costs.^7,8^ Figures indicate that insufficiently active adults spend 40% more on healthcare costs than sufficiently active adults, while potential savings on direct healthcare costs attributed to sufficient PA range from US$ 500 million to US$ 1.6 billion per year in Australia and Canada, respectively.^7,9^ The association between PA and health outcomes varies depending on context. While overall and leisure-time PA generally have positive effects on health outcomes, PA during work may have less favorable effects, a phenomenon known as the PA paradox.

Although have substantial impacts on healthcare costs among adults, it is not yet clear whether the combination of both boosts economic losses. We hypothesize that the coexistence of sleep problems and insufficient PA may have an additive effect on healthcare costs in adults. This effect would suggest that the simultaneous presence of both risk factors—poor sleep and low PA—could result in healthcare costs that exceed the sum of their individual impacts. This potential synergistic effect is particularly relevant considering the association between sleep problems and insufficient PA.^10,11^

## OBJECTIVE

The present study sought to investigate the association of combined insufficient PA and sleep problems with direct healthcare costs in adults during a four-year follow-up.

## METHODS

### Sample

This longitudinal study is part of an ongoing cohort study that began in 2010, and included adults from the Brazilian National Health Service, in the city of Bauru, state of São Paulo, Brazil. The study was approved by the Ethics Committee of the Universidade Estadual Paulista (UNESP) (process number 1046/46/01/10 date 08/24/2010).

In terms of sampling, the city of Bauru is a middle-sized city (~ 410,000 inhabitants in 2018 and human development index of 0.801) located in the most industrialized Brazilian state. In Brazil, the Brazilian National Health Service offers free-of-charge health services at all levels (primary, secondary, and tertiary) to all citizens (even foreign citizens legally living in Brazil have full access to these services). All primary care services are offered in small-to-medium-sized medical facilities, called Basic Health Units, which cover all residents living in the surrounding neighborhood. In 2010, Bauru had 17 Basic Health Units spread out in the metropolitan region of the city (only 5% of all citizens live in rural areas). The largest (number of patients) Basic Health Unit in each geographical region of the city (west, east, north, south, and center) was selected to participate in the cohort study.

**Figure 1:**
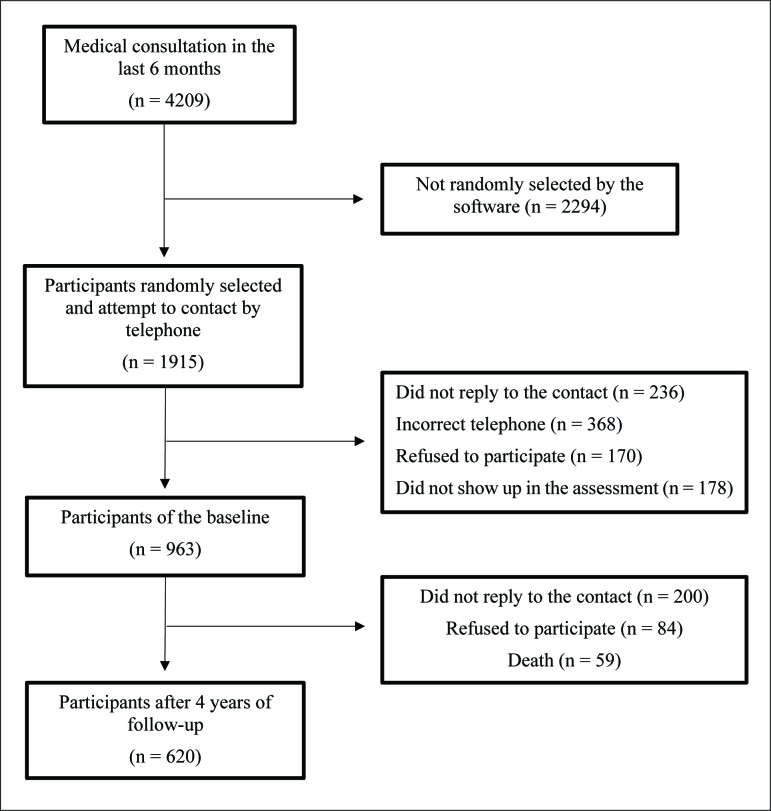
Flow diagram of participants.

Random selection was carried out in each selected Basic Health Unit according to the following inclusion criteria: i) registered for at least one year in the Basic Health Unit, ii) ≥ 50 years old, iii) an active registry (at least one consultation in the previous 6 months), and iv) signing the consent form to participate in the study. Participants who fulfilled all inclusion criteria were contacted by phone and invited to participate (face-to-face interviews and physical evaluations were scheduled for those who accepted the invitation). The minimum sample size to start this longitudinal study required 958 participants, which considered the following: i) 60% of all Brazilians exclusively used the Brazilian National Health Service, ii) error of 3.8%, iii) alpha error of 5%, and iv) sample increased by 50% due to cluster sampling. During the sampling process, 4,209 participants had at least one consultation in the last 6 months (west [n = 796], east [n = 402], north [n = 1,212], south [n = 718], and center [n = 1,081]), 1,915 (double the minimum required to protect against losses) were randomly selected/contacted (west [n = 395], east [n = 287], north [n = 416], south [n = 404], and center [n = 413]), and the final sample was composed of 963 participants (west [n = 195], east [n = 193], north [n = 193], south [n = 189], and center [n = 193]), which represents 50.2% of all contacted potential participants (Figure 1).

The minimum sample size was calculated considering expected differences in healthcare costs according to the presence of sleep problems (US$ 10.00 higher in adults with sleep problems)^6^ and insufficient PA (US$ 13.00 higher in insufficiently active adults).^12^ In all simulations for the minimum sample size, the statistical power (80%) and significance (Z = 1.95 [5%]) were standardized. Considering sleep problems, the minimum sample size required was 279 participants (93 in each group), whereas for insufficient PA, the minimum sample size was 366 participants (122 in each group). Additionally, the minimum calculated sample size was increased by 50% as a result of the inclusion of covariates in the multivariate models, and reached n = 418 and n = 549 participants for sleep problems and insufficient PA, respectively. Thus, the minimum sample size estimated for the present study was 549.

### Direct healthcare costs

Direct healthcare costs from 2010 to 2014 were assessed using medical records. The Brazilian National Health Service recommends that health professionals register all procedures performed during consultations, including but not limited to blood tests, vaccines, and drug prescriptions. Drugs prescribed during medical consultations are collected from the pharmacy in the Basic Health Unit. All healthcare services are free of charge to the patient. Each patient authorized access to the medical records and the Department of Health of Bauru provided the direct cost of each service (e.g., medical consultation, medicine taken by the patient, exams, vaccines). Total direct healthcare costs from 2010 to 2014 were calculated in the Brazilian currency (Real [R$]) and then converted to American Dollars (US$) using the official average exchange rate (adjusted for the inflation rate of the period).^8,13^

### Sleep problems

The Brazilian Portuguese version of the Mini-sleep Questionnaire was used to assess sleep problems.^14^ This questionnaire was inserted in the cohort in 2014 and comprises 10 questions assessed on a 7-point Likert scale (never-1, almost never-2, rarely-3, sometimes-4, often-5, very often-6, always-7) to evaluate different sleep aspects (sleepiness, insomnia, snoring, difficulty getting to sleep, and waking up during the night). Although the Mini-Sleep Questionnaire is a short questionnaire, it consists of two sub-scales that investigate sleep problems and daytime sleepiness. The questionnaire generates a numerical score ranging from 10 to 70 (10–24 for good sleep, 25–27 for mild sleep difficulties, 28–30 for moderate, > 30 for severe). In the present study, sleep problems were defined as a score ≥ 25.

### Insufficient PA

The Brazilian Portuguese version of the Baecke questionnaire was administered during interviews in 2010, 2012, and 2014.^15^ This questionnaire comprises 16 questions with responses rated on a 5-point Likert scale (never, seldom, sometimes, very often, and always) and addresses PA in three domains: occupational, sports participation in leisure-time, and active commuting. Only data from sports participation in leisure-time were considered for this study. We specifically selected the leisure-time domain due to its strong and well-documented association with health outcomes. This domain has been recognized as a reliable proxy for general PA levels and exhibits a direct correlation with the lifestyle factors under investigation. First, the section considers one yes/no question regarding sports participation. Participants who answer “yes” are then asked additional questions (intensity [light, moderate or vigorous], weekly volume [< 1h/week; 1–2h/week; 2–3h/week; 3–4h/week; > 4h/week], and previous time of engagement [< 1 month; 1–3 months; 4–6 months; 7–9 months; > 9 months]). In line with previous publications,^16^ sufficiently active participants were those who reported a minimum of 180 minutes per week (either 3–4 h/week or > 4h/week) of moderate-to-vigorous PA over the previous four months (either 4–6 months, 7–9 months, or > 9 months) in 2014.

### Combination of sleep problems and insufficient PA

Finally, seeking a combined variable that includes sleep problems and insufficient PA, the sample was divided into three groups: i) None (Sufficient PA + No sleep problems [n = 82]), ii) Only one (either Sufficient PA + Sleep problems [n = 12] or Insufficient PA + No sleep problems [n = 361], [n = 373]), and iii) Both (Insufficient PA + Sleep problems [n = 165]).

### Covariates

For all covariates, the baseline values were adopted. Sex, age, diagnosis of Type 2 diabetes mellitus, arterial hypertension, and any dyslipidemia were assessed during the face-to-face interview. Body mass index (BMI, kg/m^2^) was calculated using body weight (kg) and height (cm) and classified as normal (< 25 kg/m^2^), overweight (≥ 25 – < 30 kg/m^2^), and obese (≥ 30 kg/m^2^). A score for economic condition was generated using a standardized Brazilian questionnaire.^17^ Smoking status (never, former, and current smoker) was assessed during the face-to-face interview.

### Statistical analysis

Descriptive statistics included mean, median, standard deviation (SD), and 95% confidence interval (95%CI). The chi-square test was used to analyze the association between categorical data, and partial correlations were performed to analyze the relationships between variables. Analysis of covariance (ANCOVA) was used to compare healthcare costs among the three groups created by combining sleep problems and insufficient PA (models were adjusted for all covariates). The measures of effect size were expressed as eta-squared (ES-r) values. For the ANCOVA, Levene's test was used to assess the assumption of homogeneity of variances, which was considered satisfactory (P > 0.05). Repeated-measures analysis of variance (ANOVA) was used to assess changes in healthcare costs over the four years of follow-up according to the three groups. The assumption of sphericity was tested using Mauchly's test, and the Greenhouse-Geisser approach was used as a correction factor and was considered satisfactory. Interaction analysis was conducted to identify the joint effect of sleep problems and insufficient PA on healthcare costs. To this end, healthcare costs were categorized considering the 90^th^ percentile as the main outcome (an outcome of 10% was selected because odds ratio [OR] and relative risk are similar when the prevalence of the outcome is ≥ 10%) and its association with the combination of sleep problems and insufficient PA tested (P = 0.001). The OR for each category was estimated (None [OR_00_], OR = 1.0; Sufficient PA + Sleep problems [OR_01_], OR = 1.750; Insufficient PA + No sleep problems [OR_10_], OR = 1.842; Insufficient PA + Sleep problems [OR_11_], OR = 3.277). Sinergy index ([OR_11_ - 1] / {[OR_10_ - 1] + [OR_01_ - 1]}) and the proportion of the joint effects of both exposures (sleep problems and insufficient PA) attributed to interaction on healthcare costs were calculated ([OR_11_ - OR_10_ – OR_01_ + OR_00_] / OR_11_-1).^18^ A synergy index > 1.0 denotes positive additive interaction.^18^ The level of significance was set at P < 0.05 and the Stata software (StataCorp LLC., College Station, Texas, United States, version 16.0) was used to perform all analyses.

## RESULTS

A total of 963 participants were initially evaluated. After four years, 343 dropouts were recorded (due to deaths, impossible to contact the participant, desire to quit the study). Therefore, 620 participants were included in the present analysis (73.2% women; *n* = 454). Sleep problems were reported by 28.9% (95%CI: 25.2% to 32.4%) of the sample, while insufficient PA was reported by 84.8% (95%CI: 82.1% to 87.6%). From 2010 to 2014, the overall healthcare costs spent by the government on these patients reached US$ 207,174.60 (median of US$ 212.14 per patient). Participants with both sleep problems and insufficient PA presented lower economic conditions (P = 0.006), whereas the coexistence of both was associated with the female sex (P = 0.001) and obesity (P = 0.009) (Table 1).

**Table 1:** General information according to the presence of sleep problems and insufficient physical activity (Bauru, Brazil; n = 620)

		Sleep problems and Insufficient physical activity			
		None (n = 82)	Only one (n = 373)	Both (n = 165)	
Continuous		Mean (SD)	Mean (SD)	Mean (SD)	P value
Age (years)		63.9 (7.2)	65.1 (9.1)	64.2 (8.5)	0.405
Body weight (kg)		70.8 (16.1)	73.2 (14.9)	75.8 (17.3)	0.050
Height (cm)		157.9 (20.1)	157.4 (11.8)	155.7 (7.8)	0.294
EC (score)		20.1 (6.4)	18.4 (5.5)^a^	17.7 (5.5)^a^	**0.006**
Categorical (n [%])					
Sex					**0.001**
	Male	31 (37.8%)	110 (29.5%)	25 (15.2%)	
	Female	51 (62.2%)	263 (70.5%)	140 (84.8%)	
Smoking					**0.448**
	Never	37 (45.1%)	210 (56.3%)	98 (59.4%)	
	Former	39 (47.6%)	124 (33.2%)	43 (26.1%)	
	Current	6 (7.3%)	39 (10.5%)	24 (14.5%)	
BMI					**0.009**
	< 25 kg/m^2^	19 (23.2%)	66 (17.7%)	23 (13.9%)	
	25.0 – 29.9 kg/m^2^	37 (45.1%)	150 (40.2%)	62 (37.6%)	
	≥ 30.0 kg/m^2^	26 (31.7%)	157 (42.1%)	80 (48.5%)	
Diseases					
	DM (yes)	22 (26.8%)	98 (26.3%)	55 (33.3%)	0.284
	AH (yes)	61 (74.4%)	295 (79.1%)	136 (82.4%)	0.142
	DLP (yes)	27 (32.9%)	125 (33.5%)	67 (40.6%)	0.234

SD = standard deviation; BMI = body mass index; EC = economic condition; DM = diabetes mellitus; AH = arterial hypertension; DLP = dyslipidemia; a = denotes significant difference (P < 5%) compared to “None.”

Specific questions in the Mini-Sleep Questionnaire revealed that the use of hypnotic medications (r = 0.116), falling asleep during the day (r = 0.114), snoring (r = 0.093), excessive daytime sleepiness (r = 0.085), and excessive movement during sleep (r = 0.114) were associated with higher healthcare costs (Table 2). By contrast, higher intensity, weekly volume, and previous time of engagement in PA were related to lower healthcare costs.

**Table 2:** Relationship between healthcare costs and questions regarding sleep and physical activity among adults (Bauru, Brazil; n = 620)

		Dependent variable: Healthcare costs 2010-2014	
Independent variables		Partial Correlation (*r*)*	P value
Mini-sleep Questionnaire			
	Difficulty falling asleep	0.033	0.422
	Waking up too early	0.024	0.550
	Hypnotic medication use	0.116	**0.004**
	Falling asleep during the day	0.114	**0.005**
	Feeling tired upon waking up in the morning	0.058	0.150
	Snoring	0.093	**0.022**
	Mid-sleep awakenings	0.011	0.791
	Headaches on awakening	0.043	0.294
	Excessive daytime sleepiness	0.085	**0.036**
	Excessive movement during sleep	0.114	**0.005**
Baecke questionnaire			
	Intensity	-0.100	**0.013**
	Weekly volume	-0.109	**0.007**
	Previous time of engagement	-0.106	**0.009**

* Correlation adjusted by sex, chronological age, economic condition, body mass index, arterial hypertension, diabetes mellitus, and dyslipidemia.

**Figure 2:**
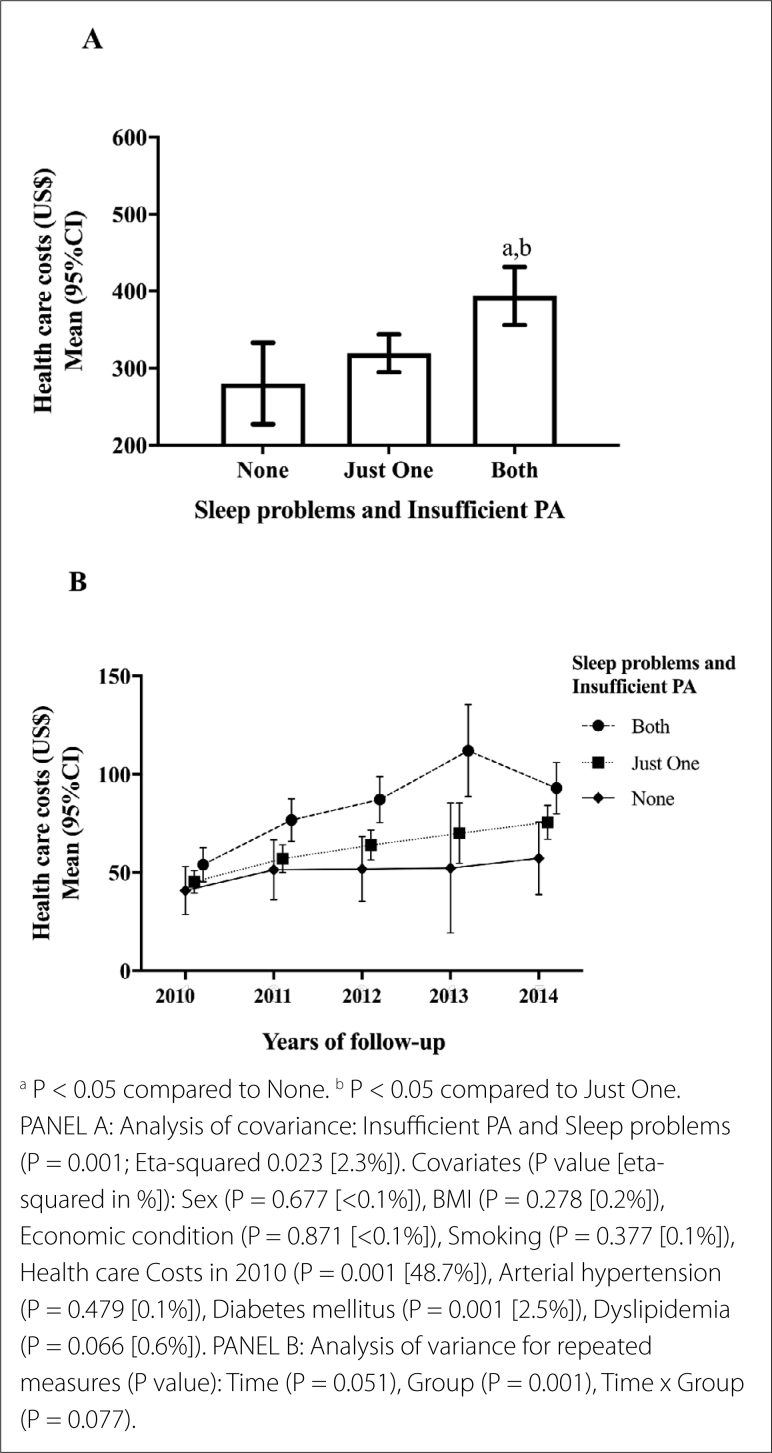
Amount of health care costs from 2010 to 2014 according to the combination of sleep problems and insufficient physical activity (Bauru, Brazil; n = 620).

The combination of sleep problems and insufficient PA explained 2.3% of all healthcare costs spent on these patients from 2010 to 2014, which directly account for approximately US$ 4,765.01. Adults who are insufficiently active and with sleep problems had the highest healthcare costs, whereas adults with either insufficient PA or sleep problems and those with neither outcome had similar costs (Figure 2, **Panel A**). In this multivariate model, the diagnosis of diabetes mellitus increased healthcare costs by approximately 2.5%.

When compared to those with no sleep problems and sufficient PA, adults with both sleep problems and insufficient PA presented higher healthcare costs throughout the follow-up period (P = 0.001) (Figure 2, **Panel B**).

Interaction analysis identified a synergy index of 1.8 (> 1.0, denoting positive additive interaction), while the proportion of the joint effects of sleep problems and insufficient PA attributed to interaction on healthcare costs reached 30.1%.

## DISCUSSION

This four-year longitudinal study established that the coexistence of sleep problems and insufficient PA increased direct healthcare costs in adults.

Sleep problems and insufficient PA were frequently identified outcomes in our participants, which is similar to previous studies^1,2,19^ and highlights the relevant problem that both outcomes present in modern society. Obesity and female were variables associated with the coexistence of sleep problems and insufficient PA. In the case of obesity, this finding is not surprising, especially because obesity is frequently associated with lower PA and a higher occurrence of sleep problems.^19,20^ Regarding sex, menopause tends to have a detrimental impact on women's body composition,^21^ while aging is related to reductions in PA in women.^9^ Thus, women over 50 years of age present a group that is potentially exposed to the harmful combination of insufficient PA and sleep problems, and thus, actions should be focused on preventing these behaviors among them.

Regarding PA, all the components assessed using the Baecke questionnaire were similarly related to healthcare costs in terms of direction and magnitude. In terms of direction, previous studies have documented the potential of PA in the mitigation of direct healthcare costs in primary care.^7,8^ Regarding magnitude, the mitigation role attributed to PA seems to be of small magnitude, below 3%.^7,8^ The low effect size attributed to the relationship between PA and healthcare costs may be explained, at least in part, by the fact that it is not a direct relationship but is mediated by the impact of PA on other variables that affect healthcare costs, such as obesity, mental health, and chronic diseases. Although the relationship between PA and healthcare costs is not large in terms of magnitude, it has been proven to be economically relevant, especially when considered at the population level.^7–9^

Regarding sleep problems, an additional analysis considering all the questions covered by the Mini-Sleep Questionnaire revealed that the most relevant questions related to healthcare costs were those that assess snoring, excessive daytime sleepiness, and the use of hypnotic medicine. The burden of hypnotic medicines on healthcare costs is not a surprise given the increased popularity of this kind of medicine among adults with sleep problems.^22^ Even so, the identification of snoring and symptoms of excessive sleepiness as determinants of healthcare costs was an interesting finding. Snoring and excessive sleepiness are frequently diagnosed symptoms in adults with obstructive sleep apnea,^20^ which is the most common sleep problem diagnosed in adults and is responsible for substantial economic losses.^5^ In fact, our study did not assess obstructive sleep apnea (which is a limitation); however, it is reasonable to believe that the observed relationships may be explained by this condition, at least in part.

Our findings revealed that the coexistence of sleep problems and insufficient PA was associated with increased healthcare costs in adults, while this impact was more tangible than that observed on the isolated manifestation of either variable (interaction analysis identified that healthcare costs were impacted in 30% due to the combination of both variables), thereby denoting both variables are potentially harmful to economic maintenance of Brazilian National Health Service. Sleep problems and insufficient PA accounted for 2.3% of all variances in healthcare costs. This figure initially seems small, but is similar to the burden of obesity on healthcare costs in Canada^7^ and of hospitalizations in Brazil, for example.^23^ Moreover, at the population level (with 36,894,000 adults ≥ 50 years old in Brazil who exclusively use the Brazilian National Health Service for health assistance^24^ and a median healthcare cost of US$ 212.14 from 2010 to 2014), and assuming there is a causal relationship in our analyses, a mitigation of 2.3% on direct healthcare costs would represent a saving of US$ 180 million in primary care services from 2010 to 2014 among adults aged ≥ 50 years.

Public health stakeholders, policymakers, and health professionals can use these results to reinforce the need for strategies to improve sleep quality and increase PA levels, especially in nations financing National Health Systems similar to Brazil (e.g., Australia, Canada, and the United Kingdom). Future clinical trials should investigate whether changes in these behaviors would mitigate healthcare costs at secondary/tertiary levels, as well as indirect costs, especially in a post-pandemic scenario during which both variables were severely affected.^25^

The study has the following limitations. Although the questionnaires used to assess both insufficient PA and sleep problems in our sample have been previously validated, widely used in epidemiological studies, and applied by trained researchers, the non-objective measures of PA and sleep may be considered relevant limitations. This aspect seems relevant because both the relationships between PA and sleep and between PA and costs seem to be affected by intensity and sleep patterns,^10,26^ which are easier to measure using objective methods. Physiological pathways linking diet and sleep problems exist, such as synthesis of serotonin and melatonin, and thus, the absence of diet control constitutes a limitation of our study. Our economic figures are probably underestimated because only primary healthcare costs were assessed, while indirect costs (e.g., absenteeism) and healthcare costs at secondary and tertiary levels (e.g., hospitalizations, surgeries) are also affected by PA and sleep problems but were not included in our analyses. Owing to the small sample size of patients with sleep problems and sufficient PA (n = 12), we were unable to split the “Only one” group. Consequently, the results should be interpreted with caution as they may not be generalizable to the broader population. Finally, although this is a longitudinal study, we are not able to infer causality for healthcare costs, mainly because sleep problems were not assessed at all time-points of data collection.

## CONCLUSION

In summary, our findings suggest that the combination of sleep problems and insufficient PA plays an important role in increasing direct healthcare costs among adults.

## References

[1] 1 Foley D, Ancoli-Israel S, Britz P, Walsh J. Sleep disturbances and chronic disease in older adults: Results of the 2003 National Sleep Foundation Sleep in America Survey. J Psychosom Res. 2004;56(5):497-502. PMID: 15172205; 10.1016/j.jpsychores.2004.02.010. 15172205

[2] 2 Kim JK, In KH, Kim JH, et al. Prevalence of sleep-disordered breathing in middle-aged Korean men and women. Am J Respir Crit Care Med. 2004;170(10):1108-13. PMID: 15347562; 10.1164/rccm.200404-519oc. 15347562

[3] 3 Barros MB de A, Lima MG, Ceolim MF, Zancanella E, Cardoso TAMO. Quality of sleep, health and well-being in a population-based study. Rev Saude Publica. 2019;53:82. PMID: 31576942; 10.11606/s1518-8787.2019053001067. PMC6763282 31576942

[4] 4 St-Onge MP, Grandner MA, Brown D, et al. Sleep Duration and Quality: Impact on Lifestyle Behaviors and Cardiometabolic Health: A Scientific Statement from the American Heart Association. Circulation. 2016;134(18):e367-86. PMID: 27647451; 10.1161/cir.0000000000000444. PMC5567876 27647451

[5] 5 Streatfeild J, Smith J, Mansfield D, Pezzullo L, Hillman D. The social and economic cost of sleep disorders. Sleep. 2021;44(11):zsab132. PMID: 34015136; 10.1093/sleep/zsab132. 34015136

[6] 6 da Silva EP, Rocha APR, Araujo MYC, et al. Sleep pattern, obesity and healthcare expenditures in Brazilian adults. Ciencia e Saude Coletiva. 2019;24(11):4103-10. PMID: 31664383; 10.1590/1413-812320182411.26972017. 31664383

[7] 7 Katzmarzyk PT, Janssen I. The economic costs associated with physical inactivity and obesity in Canada: an update. Can J Appl Physiol. 2004;29(1):90-115. PMID: 15001807; 10.1139/h04-008. 15001807

[8] 8 Codogno JS, Turi BC, Kemper HCG, et al. Physical inactivity of adults and 1-year health care expenditures in Brazil. Int J Public Health. 2015;60(3):309-16. PMID: 25680327; 10.1007/s00038-015-0657-z. 25680327

[9] 9 Gomes GAO, Brown WJ, Codogno JS, Mielke GI. Twelve year trajectories of physical activity and health costs in mid-age Australian women. Int J Behav Nutr Phys Act. 2020;17(1):101. PMID: 32778110; 10.1186/s12966-020-01006-6. PMC7418418 32778110

[10] 10 Seol J, Lee J, Park I, et al. Bidirectional associations between physical activity and sleep in older adults: a multilevel analysis using polysomnography. Sci Rep 2022;12(1):15399. PMID: 36100642; 10.1038/s41598-022-19841-x. PMC9470065 36100642

[11] 11 Alhainen M, Myllyntausta S, Pentti J, Vahtera J, Stenholm S. Concurrent changes in sleep and physical activity during the transition to retirement: a prospective cohort study. Sleep Med. 2020;68:35-41. PMID: 32028224; 10.1016/j.sleep.2019.09.009. 32028224

[12] 12 Turi BC, Codogno JS, Sarti FM, et al. Determinants of outpatient expenditure within primary care in the Brazilian National Health System. Sao Paulo Med J. 2017;135(3):205-212. PMID: 28380203; 10.1590/1516-3180.2016.0224141116. PMC10019845 28380203

[13] 13 Codogno JS, Fernandes RA, Sarti FM, Freitas Júnior IF, Monteiro HL. The burden of physical activity on type 2 diabetes public healthcare expenditures among adults: a retrospective study. BMC Public Health. 2011;11(1):275. PMID: 21542924; 10.1186/1471-2458-11-275. PMC3098169 21542924

[14] 14 Falavigna A, De Souza Bezerra ML, Teles AR, et al. Consistency and reliability of the Brazilian Portuguese version of the Mini-Sleep Questionnaire in undergraduate students. Sleep and Breathing. 2011;15(3):351-5. PMID: 20652835; 10.1007/s11325-010-0392-x. 20652835

[15] 15 Baecke JA, Burema J, Frijters JE. A short questionnaire for the measurement of habitual physical activity in epidemiological studies. Am J Clin Nutr 1982:36(5):936–42. PMID: 7137077; 10.1093/ajcn/36.5.936. 7137077

[16] 16 Fernandes RA, Zanesco A. Early physical activity promotes lower prevalence of chronic diseases in adulthood. Hypertens Res. 2010;33(9):926-31. PMID: 20574424; 10.1038/hr.2010.106. 20574424

[17] 17 Associação Brasileira de Empresas de Pesquisa - Critério de Classificação Econômica Brasil. Available from: www.abep.org. Accessed in 2023 (Oct. 4).

[18] 18 VanderWeele TJ, Knol MJ. A Tutorial on Interaction. Epidemiol Methods. 2014;3(1):33-72; 10.1515/em-2013-0005.

[19] 19 Zanuto EAC, De Limai CSM, De Araújo GR, et al. Sleep disturbances in adults in a city of Sao Paulo state. Rev Bras Epidemiol. 2015;18(1):42-53. PMID: 25651010; 10.1590/1980-5497201500010004. 25651010

[20] 20 Verbraecken J. More than sleepiness: prevalence and relevance of nonclassical symptoms of obstructive sleep apnea. Curr Opin Pulm Med. 2022;28(6):552-8. PMID: 36101923; 10.1097/mcp.0000000000000915. PMC9553267 36101923

[21] 21 Pegington M, Harvie M, Harkness EF, et al. Obesity at age 20 and weight gain during adulthood increase risk of total and premature all-cause mortality: findings from women attending breast screening in Manchester. BMC Womens Health. 2023;23(1):17. PMID: 36635680; 10.1186/s12905-023-02162-0. PMC9837983 36635680

[22] 22 Wesselhoeft R, Rasmussen L, Jensen PB, et al. Use of hypnotic drugs among children, adolescents, and young adults in Scandinavia. Acta Psychiatr Scand. 2021;144(2):100-12. PMID: 34021908; 10.1111/acps.13329. 34021908

[23] 23 Sichieri R, Do Nascimento S, Coutinho W. The burden of hospitalization due to overweight and obesity in Brazil. Cad Saude Publica. 2007;23(7):1721-7. PMID: 17572823; 10.1590/s0102-311x2007000700025. 17572823

[24] 24 Malta DC, Bernal RTI, Prates EJS, et al. Self-reported arterial hypertension, use of health services and guidelines for care in Brazilian population: National Health Survey, 2019. Epidemiol Serv Saude. 2022;8;31(spe1):e2021369. PMID: 35946670; 10.1590/ss2237-9622202200012.especial. PMC9897826 35946670

[25] 25 Dokkedal-Silva V, Morelhão PK, Moreira GA, Tufik S, Andersen ML. The next step: how sleep and physical activity can act together in the post-COVID-19 scenario. Sleep and Breathing. 2023;27(2):689-690. PMID: 35713758; 10.1007/s11325-022-02657-4. PMC9205283 35713758

[26] 26 Torres W, de Moraes Chagas LG, Fernandes RA, et al. Relationship between vigorous physical activity and health care costs among adolescents: ABCD Growth Study. BMC Pediatr. 2022;22(1):141. PMID: 35300655; 10.1186/s12887-022-03201-9. PMC8927523 35300655

